# Body mass and hibernation microclimate may predict bat susceptibility to white‐nose syndrome

**DOI:** 10.1002/ece3.7070

**Published:** 2020-12-21

**Authors:** Catherine G. Haase, Nathan W. Fuller, Yvonne A. Dzal, C. Reed Hranac, David T. S. Hayman, Cori L. Lausen, Kirk A. Silas, Sarah H. Olson, Raina K. Plowright

**Affiliations:** ^1^ Department of Microbiology and Immunology Montana State University Bozeman MT USA; ^2^ Department of Biological Sciences Texas Tech University Lubbock TX USA; ^3^ Department of Biology Centre for Forest Interdisciplinary Research (C‐FIR) University of Winnipeg Winnipeg MB Canada; ^4^ Molecular Epidemiology and Public Health Laboratory Massey University Palmerston North New Zealand; ^5^ Wildlife Conservation Society Canada Toronto ON Canada; ^6^ Wildlife Conservation Society Bronx NY USA; ^7^ Present address: Department of Biology Austin Peay State University Clarksville TN USA; ^8^ Present address: Texas Parks and Wildlife Department Nongame and Rare Species Program Austin TX USA

**Keywords:** bats, disease, evaporative water loss, hibernation energetics, microclimate, *Pseudogymnoascus destructans*, white‐nose syndrome

## Abstract

In multihost disease systems, differences in mortality between species may reflect variation in host physiology, morphology, and behavior. In systems where the pathogen can persist in the environment, microclimate conditions, and the adaptation of the host to these conditions, may also impact mortality. White‐nose syndrome (WNS) is an emerging disease of hibernating bats caused by an environmentally persistent fungus, *Pseudogymnoascus destructans*. We assessed the effects of body mass, torpid metabolic rate, evaporative water loss, and hibernaculum temperature and water vapor deficit on predicted overwinter survival of bats infected by *P. destructans*. We used a hibernation energetics model in an individual‐based model framework to predict the probability of survival of nine bat species at eight sampling sites across North America. The model predicts time until fat exhaustion as a function of species‐specific host characteristics, hibernaculum microclimate, and fungal growth. We fit a linear model to determine relationships with each variable and predicted survival and semipartial correlation coefficients to determine the major drivers in variation in bat survival. We found host body mass and hibernaculum water vapor deficit explained over half of the variation in survival with WNS across species. As previous work on the interplay between host and pathogen physiology and the environment has focused on species with narrow microclimate preferences, our view on this relationship is limited. Our results highlight some key predictors of interspecific survival among western bat species and provide a framework to assess impacts of WNS as the fungus continues to spread into western North America.

## Graphical abstract

White‐nose syndrome is an emerging disease of hibernating bats caused by an environmentally persistent fungus, *Pseudogymnoascus destructans*. We used a hibernation energetics model in an individual‐based model framework to predict the probability of survival of nine bat species at eight sampling sites across North America. These results indicate that heavier bats that roost in drier conditions, which are unsuitable for the fungus that causes white‐nose syndrome, are less susceptible to the disease. Our results highlight some key predictors of interspecific survival among western bat species and provide a framework to assess impacts of white‐nose syndrome as the fungus continues to spread into western North America.

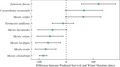

## 1 INTRODUCTION

Disease is energetically costly. In particular, the interaction between the energetic demands of an infection and the energetic demands of the environment experienced by the host can drive variation in mortality (Bonneaud et al., 2003). When a pathogen is also impacted by the environment and can persist outside the host for extended periods of time, understanding the interplay of all sides of the “disease triangle” (host, pathogen, and environment) becomes critical (Langwig et al., 2015). Furthermore, in multihost disease systems, variation in mortality often reflects host physiological adaptations and the susceptibility of the host to disease (Dobson, 2004; Langwig et al., 2016).

Environments that require low energetic input by the host in the absence of disease may become unsuitable if conditions facilitate extensive pathogen growth or transmission (Nowakowski et al., 2016). For example, *Pseudogymnoascus destructans*, the pathogenic fungus that causes white‐nose syndrome (WNS) in hibernating bat species, thrives in cool, moist conditions (Langwig et al., 2012; Verant et al., 2012). Hibernacula that align with these conditions are the least energetically demanding environments for healthy bats (Geiser, 2004). Throughout hibernation, bats alternate between periods of reduced metabolic rate and body temperature (i.e., torpor) and periods of euthermia. Although the concept of “optimal” environments for hibernation is debated, it is generally accepted that healthy bats can reduce their fat loss over winter by selecting microclimates that reduce arousal frequency (Boyles, Boyles et al., 2017; Boyles, Dunbar et al., 2007; Boyles et al., 2020; Humphries et al., 2003; Nowack et al., 2019). As bats drop body temperature to near‐ambient temperature during torpor, minimum torpid metabolic rate is used at colder ambient temperatures until hibernacula reach a species’ minimum defended temperature (Geiser, 1988, 2004). The need to arouse to euthermic body temperatures during hibernation has been attributed to many factors, including the need to replenish water lost through evaporation (Ben‐Hamo et al., 2013). Humid environments decrease evaporative water loss, increase torpor bout duration, and decrease energy expenditure (McGuire et al., 2017; Thomas & Cloutier, 1992). However, the populations and species suffering the highest WNS mortality rates hibernate in humid environments (Langwig et al., 2012) that allow for extensive fungal growth. In sum, the environments that once provided low energetic demands for the host could also lead to high fungal burdens and the demise of bat populations as the fungus moves across species’ ranges.

Many hibernating bat species in North America are often found roosting in drier conditions than the populations that have been well‐studied in eastern North America (Baerwald, 2017; Gillies et al., 2014; Grieneisen, 2011; Jagnow, 1998; Klüg‐Baerwald et al., 2017; Klüg‐Baerwald & Brigham, 2017; Kuenzi et al., 1999; Neubaum et al., 2006). Inter‐ and intraspecific studies have shown that bats that roost in drier conditions have mechanisms to decrease rates of evaporative water loss when exposed to extreme conditions (Boratyński et al., 2015; Gearhart et al., 2019; Klüg‐Baerwald & Brigham, 2017). We surmise that these species may be less susceptible to the impacts of WNS, as drier hibernacula could limit fungal growth (Marroquin et al., 2017), and these species may have the mechanisms to reduce water loss associated with these conditions. Variation in hibernaculum microclimates facilitates a natural experiment to test the effects of environmental conditions, host physiology, and pathogen growth on mortality. Furthermore, we can assess if previously suitable conditions for minimum energy consumption for the host become unsuitable in the presence of a pathogen due to increased fungal growth and thus higher susceptibility to disease.

Multiple studies have investigated factors that are important to WNS survival, including body mass (Haase et al., 2019), prehibernation fat stores (Cheng et al., 2019), physiological mechanisms for resistance to infection (Auteri & Knowles, 2020; Hoyt et al., 2015), and hibernaculum microclimate (Langwig et al., 2012; Verant et al., 2014). However, these studies have related these covariates independently and often within a single species. When discussing WNS survival across species, studies have focused on species that hibernate in a narrow range of microclimate conditions, which can miss critical aspects of survival. For example, Langwig et al. (2012) presented variation in survival with WNS across species as a function of hibernaculum relative humidity and temperature. However, the minimum measured relative humidity was 90%, whereas hibernaculum environments experienced by North American bat species susceptible to WNS range from 20%–100%.

As WNS has the greatest impacts in eastern North America, most research on the interplay between host and pathogen physiology and hibernaculum microclimate has focused on eastern bat species. These species roost in stable microclimates, and thus, we have a limited view on how interspecific variation in bat physiology may influence disease dynamics of WNS. Here, we combine field data with mathematical modeling to test the relative importance of bat morphology, bat physiology, and hibernation microclimate in explaining variation in survival from WNS across nine species roosting in a range of hibernaculum conditions. We applied the Haase et al. (2019) hibernation energetics model in an individual‐based model framework to assess survival as a function of species‐specific bat characteristics, hibernaculum microclimate, and fungal growth rate at eight sampling sites across North America. Hayman et al. (2016) and Haase et al. (2019) incorporated the effect of fungal growth on fat consumption and evaluated the sensitivity of the model to bat morphometric parameters. Our model incorporates the main physiological variables believed to drive variation in WNS survival across species and assess the relative importance of these physiological variables.

## 2 METHODS

We used a combination of field data and energetic modeling to predict the probability of survival during hibernation with WNS for nine bat species: *Corynorhinus townsendii*, *Eptesicus fuscus*, *Myotis ciliolabrum*, *Myotis evotis*, *M. lucifugus*, *Myotis thysanodes*, *Myotis velifer*, *Myotis volans*, and *Perimyotis subflavus* at eight sites located in Oklahoma, Utah, Nevada, Montana, Colorado, and Oregon. Our hibernation energetics model uses mathematical equations to calculate total fat consumption during each phase of a torpor–arousal cycle as a function of bat morphology and physiology, hibernaculum microclimate, and winter duration. Recent work has also incorporated growth of *P. destructans* into the model (Haase et al., 2019; Hayman et al., 2016), which allows prediction of fat consumption given fungal growth during hibernation. The full hibernation model, including methods to derive parameters, model sensitivity, and model validation, is presented in Haase et al. (2019).

**Figure 1:**
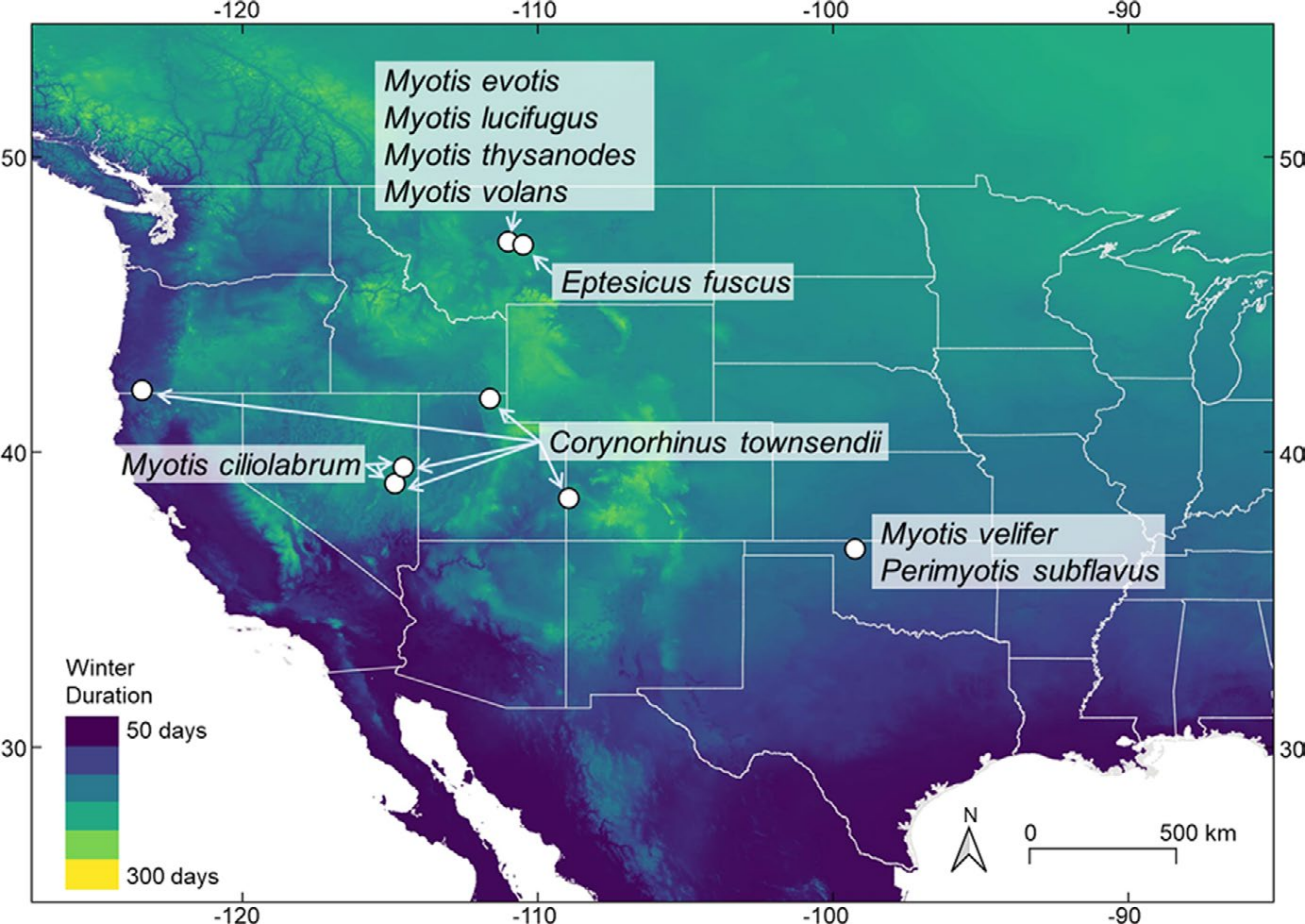
Predicted winter duration (in days) and sites of sampled hibernaculum with species sampled at each site

We predicted the probability of survival with WNS for each species in an individual‐based model framework. Microclimate data (temperature, water vapor deficit), bat morphometrics (body mass), and bat physiological characteristics (torpid metabolic rate, evaporative water loss) were collected from the field from eight sites (Table 1, Figure 1; [Link], [Link]). We predicted winter duration at each sampling site given estimates from Hranac et al. (unpublished data, CRH, CGH, NWF, JCM, CLL, LPM, SHO,) and assumed that all individuals at that site would enter into and emerge from hibernation associated with those predictions to remove the effect of intraspecific variation in hibernation duration. Finally, we estimated hourly fungal growth rate as a function of hibernaculum temperature and water vapor deficit given equations described by Hayman et al. (2016).

**Table 1:** Information for each hibernaculum site, including predicted winter duration (with 95% confidence intervals; CRH unpublished data) and microclimate conditions

State	Site type	Species present	Winter duration (days)	Across all data loggers			At recorded bat locations		
				T_a_ (°C)	RH (%)	dWVP (kPa)	T_a_ (°C)	RH (%)	dWVP (kPa)
Montana	Cave	*Myotis evotis*	184 ± 12	5.8 (7.0)	90.2 (19.4)	0.17 (0.42)	6.8 (5.2)	91.1% (0.9)	0.13 (0.01)
		*Myotis lucifugus*							
		*Myotis thysanodes*							
		*Myotis volans*							
Montana	Cave	*Eptesicus fuscus*	199 ± 16	4.1 (6.1)	78.6 (19.4)	0.27 (0.25)	5.4 (0.6)	72.67 (20.1)	0.21 (0.20)
Nevada	Mine	*Corynorhinus townsendii*	169 ± 12	6.5 (4.0)	47.6 (17.1)	0.54 (0.27)	7.9 (1.7)	79.3 (10.3)	0.22 (0.11)
		*Myotis ciliolabrum*							
Nevada	Mine	*Corynorhinus townsendii*	172 ± 12	13.4 (7.1)	42.9 (16.2)	1.09 (0.85)	7.6 (1.6)	65.3 (4.8)	0.39 (0.06)
		*Myotis ciliolabrum*							
Oklahoma	Cave	*Myotis velifer*	134 ± 18	10.2 (5.2)	88.2 (18.1)	0.17 (0.38)	10.5 (4.3)	76.6 (4.9)	0.24 (0.12)
		*Perimyotis subflavus*							
Colorado	Mine	*Corynorhinus townsendii*	152 ± 23	5.2 (5.2)	78.6 (19.4)	0.21 (0.27)	10.0 (6.9)	57.1 (18.4)	0.61 (0.49)
Oregon	Cave	*Corynorhinus townsendii*	125 ± 14	5.7 (2.8)	95.7 (12.6)	0.06 (0.21)	6.5 (1.2)	87.0 (5.6)	0.12 (0.05)
Utah	Cave	*Corynorhinus townsendii*	172 ± 16	10.6 (9.7)	68.8 (19.0)	0.59 (0.61)	2.3 (5.9)	99.8 (1.1)	0.00 (0.00)

Note

Temperature (T_a_), relative humidity (RH), and water vapor deficit (dWVP) mean and standard deviation (in brackets) are reported for all data loggers within the hibernaculum and at the specific location where bats of any species were observed.

We ran the hibernation energetics model for 100 independent bats within 100 variations of hibernaculum microclimate for 10,000 bat‐runs of fat expenditure per species. We characterized a hibernaculum microclimate environment by randomly selecting temperature and relative humidity values from normal distributions fitted to the mean and standard deviation from our measured microclimate data from the sampling location (where bats were found roosting) within the site associated with that species (Table 1). The randomly sampled temperature and relative humidity combination represented the “winter” condition for each individual bat‐run. We used relative humidity, rather than water vapor deficit to characterize the hibernaculum microclimate in order to prevent impossible water vapor deficit and temperature combinations. We then converted to water vapor deficit (difference in water vapor pressure [kPa] between air and saturation at measured air temperature and relative humidity) once the microclimate was characterized using equations from Campbell and Norman (1998). For species found at multiple sites, we combined the data across sites, as previous analyses indicate no site‐specific variation in morphology or physiology (McGuire et al, unpublished data, LPM, NWF, YAD, CGH, KAS, CKRW, SHO, CLL). We defined the specific microclimate conditions measured by the data loggers that were closest to the roosting locations of each species at our sampling sites. We assume that these conditions represent the conditions most bats experienced during hibernation at these sites, though note that bats could move freely around the hibernaculum. Using our measured morphometric and physiological data for each species, we randomly selected body mass from a normal distribution fitted to the mean and randomly selected minimum torpid metabolic rate from a lognormal distribution fitted to the mean‐log and standard deviation (Table 2). As we already understand that fat is a critical factor required for survival (Cheng et al., 2019), we held the proportion of fat constant across species to test for the relative influence of other factors and assumed prehibernation fat stores were 25% of body mass and lean mass was 65% (L. P. McGuire, pers. comm.).

**Table 2:** Mean morphometric and physiological parameters, including body mass, minimum mass‐specific torpid metabolic rate (TMR), the temperatures for TMR measurements, and mass‐specific evaporative water loss

Species	Body mass (g)	*N*	Minimum TMR (ml O_2_ hr^−1^ g^−1^)	Temperatures for minimum TMR measurements	*N*	Evaporative water loss (mg H_2_O hr^−1^ g^−1^)	*N*
*Corynorhinus townsendii*	11.0 (1.0)	157	0.04 (0.04)	5°C, 8°C	57	0.57 (0.37)	52
*Eptesicus fuscus*	19.3 (2.2)	5	0.03 (0.01)	2°C, 5°C, 8°C, 10°C	5	0.58 (0.41)	5
*Myotis ciliolabrum*	5.7 (0.4)	33	0.03 (0.02)	2°C, 5°C, 8°C, 10°C	16	0.58 (0.37)	14
*Myotis evotis*	7.4 (0.6)	201	0.05 (0.03)	5°C, 8°C, 10°C	17	1.16 (0.50)	9
*Myotis lucifugus*	7.7 (0.7)	163	0.03 (0.03)	2°C, 5°C, 8°C	86	0.85 (0.25)	86
*Myotis thysanodes*	9.4 (1.0)	43	0.03 (0.01)	5°C, 8°C, 10°C	6	1.06 (0.42)	8
*Myotis velifer*	15.1 (1.4)	182	0.05 (0.04)	5°C, 8°C, 10°C	8	0.90 (0.12)	10
*Myotis volans*	8.7 (0.9)	83	0.04 (0.03)	5°C, 8°C, 10°C	6	0.90 (0.42)	9
*Perimyotis subflavus*	6.9 (0.7)	79	0.02 (0.02)	8°C, 10°C	11	1.04 (0.53)	15

Note

The number of samples (*N*) and standard deviation (in brackets) for each parameter are also reported.

We ran the hibernation energetics model for the predicted winter duration for each bat and determined if survival occurred by comparing the fat mass going into and emerging from hibernation; if the starting fat mass was not expended during the hibernation period, then survival occurred. We calculated the probability of survival for each species over each of the 100 winter runs as the total number of bats that survived over the population of 100—that is, if 65 out of the 100 bats did not expend all fat stores before winter concluded, the survival probability for that population was 65%. As the hibernation energetics model calculates the number of days until total fat expenditure, we also calculated the difference between predicted winter duration and predicted winter survival for each bat and calculated the mean and standard deviation for each species. This difference allows us to visualize the variation in survival for each species, as well as how much longer each species could potentially survive in the hibernaculum environment postspring emergence as a proxy of body condition on emergence.

We fit a linear model to the probability of survival with body mass, mass‐specific torpid metabolic rate, mass‐specific evaporative water loss, hibernaculum temperature, and hibernaculum water vapor deficit as predictors. Because mass‐specific evaporative water loss and hibernaculum water vapor deficit are mechanistically linked, we fit two models, one with mass‐specific evaporative water loss and one with hibernaculum water vapor deficit, and selected the model with the highest adjusted *R^2^* value. We then calculated the partial correlation coefficient (PCC; Baba et al., 2004) and squared semipartial correlation coefficient (SPCC; Kim, 2015) for each covariate. Both the PCC and the SPCC measure the correlation between a covariate of question (e.g., body mass) and the dependent variable (i.e., probability of survival), with the effect of the other covariates removed in some form. The SPCC compares the unique variation of a single covariate with the dependent variable, without the influence of the other variables on the covariate (Kim, 2015)—the effect of the other variables on the covariate in question is removed. The PCC compares the unique variation of the covariate to the unique variation of the dependent variable (Baba et al., 2004)—the effect of the other variables is removed from both the dependent variable and the covariate. The squared SPCC reflects the variation in the dependent variable explained by the covariate in question, not including the variance in the dependent variable explained by other covariates. Therefore, we determined which variables (hibernaculum temperature and water vapor deficit, mass‐specific minimum torpid metabolic rate, mass‐specific evaporative water loss, and body mass) explained the most variation in survival with WNS and fulfilled our predictions.

We were also interested in how these variables differed among species and whether there was any significant variation in survival during hibernation with WNS. We first performed a multiple comparisons Kruskal–Wallis rank‐sum test of differences (Daniel, 1990) to test for pairwise differences between species. We also calculated Tukey's honest significant differences (Tukey, 1949) to determine pairwise differences in body mass, mass‐specific torpid metabolic rate, and mass‐specific evaporative water loss between species. We assume that if the species share the same predictor traits, we predict there will be no significant differences in survival.

## 3 RESULTS

**Figure 2:**
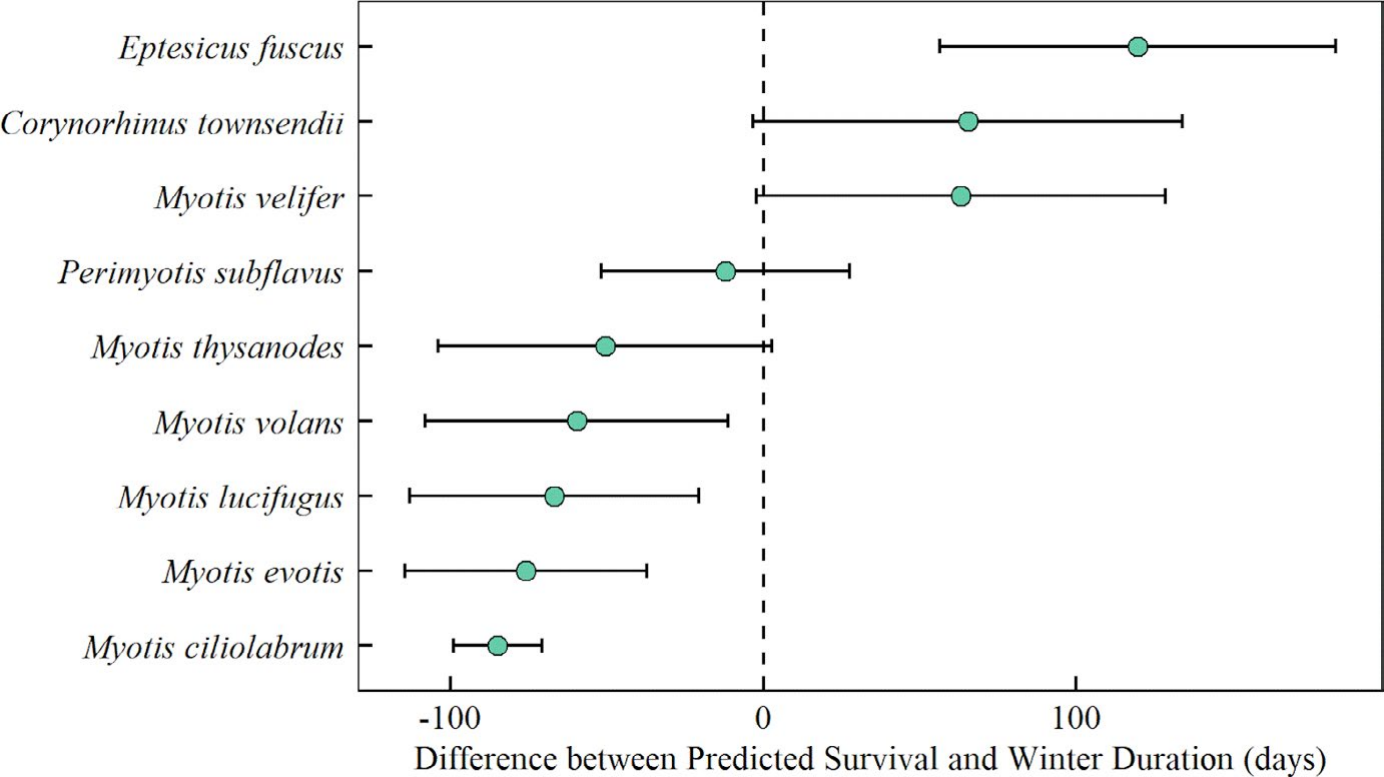
Difference between predicted days until fat exhaustion and predicted winter duration (with standard deviation error bars). There were significant species differences in survival (Table S1). All small *Myotis* species, including *M. ciliolabrum*, *M. evotis*, *M. lucifugus*, *M. thysanodes*, and *M. volans*, did not exhibit differences in predicted survival at our measured sampling sites. Additionally, *M. velifer* did not differ from *C. townsendii* nor *E. fuscus* in predicted survival, but these three did differ from the rest of the *Myotis* species

There were species differences in survival during hibernation with WNS predicted at our sampling sites (*X* ^2^ = 529.6, *df* = 8, *p*‐value < .001, critical difference = 117.53; Table S1). There were no differences in survival between *M. velifer* and *E. fuscus* (observed difference = 90.10 days) nor *M. velifer* and *C. townsendii* (observed difference = 82.38 days). All three species survived winter with WNS for most, if not all, of the modeled environmental conditions (Figure 2). *E. fuscus* survived in all scenarios and contained enough fat to hibernate for an additional 120 days in hibernation past the predicted spring emergence date. *M. velifer* and *C. townsendii* survived for most of the scenarios, but not all. Both species had enough fat to survive an additional month in hibernation, on average. There was no difference in survival among all small *Myotis* species, including *M. ciliolabrum, M. evotis*, *M. lucifugus*, *M*. *thysanodes*, and *M*. *volans*. These five species did not survive in most of the microclimate scenarios and model predictions showed mortality between 3 and 4 months of hibernation. *M. thysanodes* had high mortality, but was predicted to survive in some microclimate scenarios. *P. subflavus* had a wide range of survival across microclimate scenarios, but on average, did not have enough fat to survive hibernation. Between species, there were no patterns of pairwise differences in body mass, mass‐specific torpid metabolic rate, mass‐specific evaporative water loss, or hibernaculum microclimate, as we predicted (Table S1). In other words, similar pairwise trait patterns did not appear among the bats predicted to survive or succumb to WNS.

When determining which morphometric, physiological, or microclimate variables were strong predictors, we found hibernaculum temperature and water vapor deficit, mass‐specific minimum torpid metabolic rate, mass‐specific evaporative water loss, and body mass explained 58% of the variation in winter survival with WNS (Table 3). Hibernaculum water vapor deficit explained more variation (9.0%) than mass‐specific evaporative water loss (1.5%) and thus the model with water vapor deficit explained more overall variation in survival (adjusted *R* ^2^ = 0.89) compared to mass‐specific evaporative water loss (adjusted *R* ^2^ = 0.75). Body mass (*p*‐value = 0.004) and hibernaculum water vapor deficit (*p*‐value = 0.043) were the only significant predictors of the probability of survival through hibernation with WNS (Table 3). When assessing each covariate alone, water vapor deficit (8.27%) and body mass (47.0%) explained over half of the variation in survival, while the other covariates explained less than 4% (Table 3). According to the estimated parameter values, high humidity and smaller body mass resulted in decreased survival with WNS.

**Table 3:** Parameter estimates, standard errors (*SE*), *p*‐values, squared semipartial correlation coefficient (SPCC), and partial correlation coefficient (PCC) of covariates describing survival with white‐nose syndrome in nine bat species

Parameter	Estimate	*SE*	*p*‐value	SPCC	PCC
T_a_ (°C)	−0.001	0.05	0.98	0.01	0.20
dWVP (kPa)	0.156	0.05	0.04	0.09	0.71
Body mass (g)	0.278	0.05	0.01	0.47	0.93
Mass‐specific TMR (ml O_2_ hr^−1^ g^−1^)	−0.005	0.05	0.92	0.01	0.01
Mass‐specific EWL (mg H_2_O hr^−1^ g^−1^)	—	—	—	0.02	0.29

Note

Squared SPCC values indicate the contribution of each variable to describing variation in survival. Hibernaculum temperature (T_a_), water vapor deficit (dWVP), body mass, mass‐specific torpid metabolic rate (TMR), and mass‐specific evaporative water loss (EWL) were measured across eight sampling sites.

## 4 DISCUSSION

Unraveling the complex interactions between the host, pathogen, and environment (the “disease triangle”) helps us understand the influence of external pathogens on hibernation physiology and winter survival. In hibernating bats of North America, WNS is a disease where the environment clearly influences both the host and the pathogen. We used the natural physiological and environmental host diversity of western bats in order to address the importance of each of these factors on bat overwintering mortality. Our application of hibernation modeling to empirical field data allows for the estimation of survival from WNS across multiple species that use different hibernation conditions. We show that hibernaculum water vapor deficit and host body mass are likely strong predictors of susceptibility to WNS. Our results indicate that body mass is the strongest predictor for survival with WNS and we note pairwise differences between species with differing body mass. We found that species roosting in drier environments are predicted to have higher survival than those that roost in hibernacula at or near saturation. Saturated conditions, though less energetically costly for hibernation by healthy individuals, lead to greater fungal growth rates and thus higher mortality (Haase et al., 2019; Langwig et al., 2012), which suggests a trade‐off between water conservation and fat conservation in bats infected with WNS.

**Figure 3:**
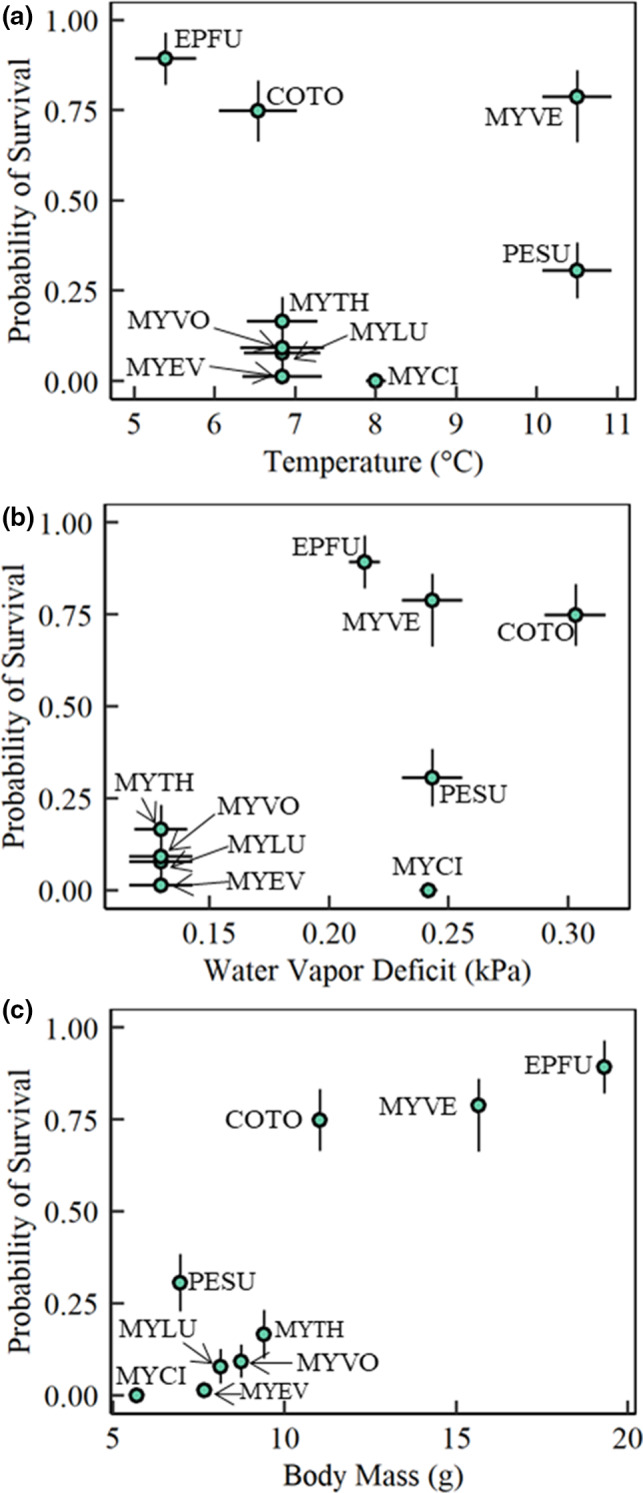
Predicted survival over hibernation with white‐nose syndrome in response to (a) hibernaculum temperature (°C). (b) Hibernaculum water vapor deficit (kPa). (c) Body mass (g) of nine species: *Corynorhinus townsendii* (coto), *Eptesicus fuscus* (epfu), *Myotis ciliolabrum* (myci), *Myotis evotis* (myev), *Myotis lucifugus* (mylu), *Myotis thysanodes* (myth), *Myotis velifer* (myve), *Myotis volans* (myvo), and *Perimyotis subflavus* (pesu)

Taken together, our results suggest that the importance of body mass for survival from previous work may be related to the amount of surface area available for evaporative water loss. For example, though *E. fuscus* is a much larger bat than *M. fuscus*, it does not necessarily mean they have proportionally more fat than *Myotis* species. Instead, *Myotis* have greater rates of evaporative water loss per unit of body mass than larger bats due to the greater surface area per unit volume, which is an important component to arousal rates in hibernating bats (Ben‐Hamo et al., 2013; McGuire et al., 2017). The fact that small species which roost in dry hibernacula (i.e., *Myotis ciliolabrum*) can have low survival (Figure 3) indicates that greater body mass, and therefore less surface area relative to body size, is still imperative for survival.

The relationship between predicted survival and water vapor deficit within the hibernaculum demonstrates how microclimate can influence WNS susceptibility. Our results are consistent with results from empirical studies that find an association between population declines and humid environments (Langwig et al., 2012). Suboptimal environments for fungal growth are potentially unsuitable environments for bat hibernation given each species physiology and behavior. However, we found that if bats have the predisposition, and thus adaptations, for successfully hibernating in unsaturated environments, then these individuals may survive hibernation with WNS. Therein lies a trade‐off between fat conservation and water conservation—if bats are adapted to roosting in saturated conditions, then drier environments would still result in high mortality due to increased evaporative water loss, with or without the impact of the fungus. Therefore, we cannot explicitly state that suboptimal environments for the fungus would result in higher rates of survival, but rather need to take a holistic approach to understanding the specific hibernation strategies of each bat species, recognizing that intraspecific differences can exist (e.g., Klüg‐Baerwald and Brigham, 2017). Additionally, our models only predict survival based on sufficient fat stores, but do not consider fat stores posthibernation, when remaining stores are necessary for flight and reproduction, nor the energy required for the costly inflammatory response to fight off infection (Fuller et al., 2020).

As many of the species studied here are widespread across North America, variation in hibernaculum microclimate selection may result in differential selective pressures acting within species and consequential variation in adaptations to evaporative water loss. In previous work (McGuire et al., in review), we did not find evidence of variation among populations spread across biomes or large geographic distances, concluding that physiology does not differ when bats have access to their preferred conditions. But when pushed outside preferred conditions, and under more extreme experimental conditions, differences among populations may become apparent. *E. fuscus* populations from more arid regions had lower rates of evaporative water loss than those from more mesic regions when measured in dry (0% relative humidity) conditions (Klüg‐Baerwald & Brigham, 2017). Additionally, *E. fuscus* maintains more variable torpid metabolic rates in northern latitudes compared to southern latitude conspecifics at temperature below the minimum defended temperature, suggesting a continuum in thermoregulatory responses and minimum defended temperatures to hibernaculum environments (Dunbar & Brigham, 2010). However, these species vary in microclimate selection, while other species, such as *M. lucifugus*, are noticeably more selective in their hibernacula (Brack, 2007). *M. lucifugus* tends to roost in hibernacula that are at or very close to saturation with stable temperatures (Grieneisen, 2011).

When determining the conditions that may favor survival, it is important to consider how species and populations are adapted. It may be generally true that low humidity sites are detrimental to the fungus, and therefore, some species may experience high survival in low humidity, but that is only true for those species that are adapted to those conditions. For instance, we predicted survival for five species that were found in dry conditions (Figure 3b), but only three of them are predicted to have high survival (> 75%). Previous measurements of these species indicate that those that have higher survival also have low rates of evaporative water loss. Other species, such as *P. subflavus*, were found to have high rates of evaporative water loss, and thus are not predicted to survive in these conditions. In the case of *P. subflavus*, we sampled a population at the extreme edge of the species’ distribution and therefore may not have access to the preferred hibernating conditions. On the other hand, *M. ciliolabrum* had low rates of evaporative water loss but was still predicted to have low survival due to its low body mass, which indicates the relative importance of each of these factors. Other work (Hranac et al., in prep) may indicate different predictions for these species across their distribution, signifying the importance of local microclimate selection on survival.

Contrary to previous work that indicated a positive relationship between hibernaculum temperature and fungal loads (Langwig et al., 2016), hibernaculum temperature did not affect predicted survival in our model. Previous work relating hibernaculum temperature to WNS persistence focused solely on eastern bat species (Brack, 2007; Grieneisen et al., 2015; Langwig et al., 2012), which tend to roost in hibernacula with stable temperatures and are at saturation. We propose that this difference is due to the wider range of hibernation behaviors and physiology in the species and populations we included in our analysis. For instance, *M. lucifugus* is a broad‐ranging species, yet has very specific microclimate requirements that dictate survival from WNS (Grieneisen et al., 2015; Johnson et al., 2014). On the other hand, we also included *E. fuscus* and *C. townsendii* in our analysis, which are species that select a broader range of microclimates for hibernation that results in variation in hibernation physiology. These species may not be as impacted by hibernaculum temperature compared to the influence of saturation of the hibernaculum. Additionally, we surmise that because there was no influence of temperature on survival, torpid metabolic rate also had no impact. Further work to understand variation in physiology and behavior is warranted, specifically in the adaptations that may or may not influence bat survival through hibernation with WNS (e.g., low evaporative water loss).

We modeled nine bat species at eight overwintering sites and the model framework could be extended to different species and hibernacula at different latitudes and longitudes as data become available. Until then, extrapolation to sites with parameters that fall outside those of our study sites should be done with caution. Future improvements to our model could include interspecific differences in hibernation behavior across species; for example, one could incorporate clustering behavior and its effect on heat loss and evaporative water loss (Boratyński et al., 2015; Boyles et al., 2008). However, there is evidence that clustering can cause additional or partial arousals in individuals when a neighbor arouses (Hayman et al., 2017), suggesting that clustering may increase fat expenditure in colonies with WNS (Langwig et al., 2012). Solitary roosting has also been observed in many western bat species, where individuals roost alone in crevices and talus slopes (Neubaum et al., 2006). Solitary roosting most likely reduces fungal transmission, but may change microclimates experienced by individual bats. For instance, in the hibernacula where both *C. townsendii* and *M. ciliolabrum* were observed, *C. townsendii* roosted on mine walls in clusters, whereas *M. ciliolabrum* roosted solitarily in tight crevices. We suspect that the temperatures and water vapor deficits experienced by these crevice‐roosting bats may not be represented by the microclimate measurements of our sensors. More information is required on how clustering or solitary roosting may alter hibernation behavior and physiology and ultimately survival from WNS.

Most WNS research has focused on eastern bat species that roost in stable microclimate conditions within hibernacula. Thus, we have a limited view not just on hibernation physiology, but how physiology may influence disease impacts from WNS. Predicting survival from WNS is complicated as physiology and morphology vary across species, and predictions made based on the physiology and morphology of one species (i.e., *M. lucifugus*) may not translate well to others. As we show in our analyses, this model species consistently has low survival due to WNS < while other species, such as *E*. fuscus or *C. townsendii*, were predicted to have high survival. Predictions have to consider the preferences and adaptations of populations and species to microclimates, and take into account variation both across and within species to WNS. Our research aimed to untangle how variation in hibernation behavior and physiology and hibernaculum humidity influences survival with WNS. Our results highlight the key predictors of interspecific survival among western bat species and provide a framework to assess impacts of WNS. We can use our findings to better understand the complex dynamic between host and pathogen physiology with the environment, and how unraveling these relationships can better predict impacts of WNS as it spreads across North America.

## CONFLICT OF INTEREST

The authors declare no conflict of interest.

## Author Contribution

**Catherine G. Haase:** Conceptualization (lead); Data curation (lead); Formal analysis (lead); Investigation (equal); Methodology (lead); Validation (lead); Visualization (lead); Writing‐original draft (lead). **Nathan W. Fuller:** Data curation (equal); Investigation (equal); Writing‐review & editing (equal). **Yvonne A. Dzal:** Data curation (equal); Writing‐review & editing (equal). **C. Reed Hranac:** Formal analysis (equal); Writing‐review & editing (equal). **David T. S. Hayman:** Conceptualization (equal); Funding acquisition (equal); Writing‐review & editing (equal). **Cori L. Lausen:** Data curation (equal); Funding acquisition (equal); Writing‐review & editing (equal). **Kirk A. Silas:** Data curation (equal). **Sarah H. Olson:** Conceptualization (equal); Funding acquisition (lead); Project administration (lead); Resources (lead); Supervision (equal); Writing‐review & editing (equal). **Raina K. Plowright:** Conceptualization (equal); Funding acquisition (lead); Project administration (equal); Resources (equal); Supervision (equal); Writing‐review & editing (equal).

## Supporting information

Supplement S1

Supplement S2

## Data Availability

Morphological, physiological, and microclimate data are available on Dryad: https://doi.org/10.5061/dryad.wh70rxwm5
