# Supplementary material for: Body mass and hibernation microclimate may predict bat susceptibility to white‐nose syndrome
Source: Ecol Evol. 2020 Dec 21;11(1):506–15. doi: 10.1002/ece3.7070 (PMC7790633; doi:10.1002/ece3.7070)
Supplement: Supplementary file 1 — Supplement S1 [file ECE3-11-506-s001.pdf]

Body mass and hibernation microclimate may predict bat susceptibility to white-nose syndrome

Catherine G. Haase, Nathan W. Fuller, Yvonne A. Dzal, C. Reed Hranac, David T. S. Hayman, Cori L. Lausen, Kirk A. Silas, Sarah H. Olson, and Raina K. Plowright

11/11/2020

About

This code is associated with "Body mass and hibernation microclimate may predict bat susceptibility to white-nose syndrome", published in Ecology and Evolution. The data are available on Dryad (https://doi.org/10.5061/dryad.wh70rxwm5).

We used a hibernation energetics model in an individual-based model framework to calculate the probability of survival over hibernation with white-nose syndrome (WNS) for each species. The hibernation energetics model predicts fat consumption as a function of bat morphological and physiological characteristics, hibernaculum microclimate, and fungal growth. We used data collected from the field to parameterize the model. The full hibernation model, methods to derive parameters, model sensitivity, and model validation are presented in Haase et al. (2019).

First install `batwintor` from Github with associated branch.

```
devtools::install_github("cReedHranac/batwintor", ref = "Testing")
library(batwintor)
```

Summarize Morphometric & Physiological Data

To parameterize the individual-based hibernation energetics model, we randomly selected body mass and torpid metabolic rate from distributions fit to the mean and standard deviation of our field data. We first checked that the field data met the assumption of a normal distribution with a Shapiro-Wilk Test of Normality and found that our variables follow a normal/log-normal distribution.

Read in data files.

```
#Morphometrics data file
morph <- read.csv("Haase_morphometrics.csv")

#Respirometry data file
resp <- read.csv("Haase_respirometry.csv")

#Model parameter data file from the batwintor package
params <- read.csv("parameters.csv")
```

Now we can create a data frame of the mean and standard deviation for each species.

```
library(tidyverse)

#Calculate mean and standard deviation of body mass
mass.df <- morph[!is.na(morph$capture.mass),] %>%
```

```
            group_by(species) %>%
            summarise_at(vars(capture.mass), funs(mean, sd, length))
IBM <- data.frame(mass.df)
colnames(IBM) <- c("Species", "Mass.Mean", "Mass.SD", "Mass.N")

#We need to log for a log-normal distribution
resp$tmr.mw.g.LN <- log(resp$tmr.mw.g)

#Calculate mean and standard deviation of TMR
TMR.df <- resp[!is.na(resp$tmr.mw.g),] %>%
            group_by(species) %>%
            summarise_at(vars(tmr.mw.g,tmr.mw.g, tmr.mw.g,tmr.mw.g.LN),
                         funs(mean, sd, length))
IBM$TMR.Mean  <- TMR.df$tmr.mw.g_mean[match(IBM$Species,TMR.df$species)]
IBM$TMR.SD    <- TMR.df$tmr.mw.g_sd[match(IBM$Species,TMR.df$species)]
IBM$TMR.N     <- TMR.df$tmr.mw.g_length[match(IBM$Species,TMR.df$species)]
IBM$TMR.LMean <- TMR.df$tmr.mw.g.LN_mean[match(IBM$Species,TMR.df$species)]
IBM$TMR.LSD   <- TMR.df$tmr.mw.g.LN_sd[match(IBM$Species,TMR.df$species)]
IBM$TMR.LN    <- TMR.df$tmr.mw.g.LN_length[match(IBM$Species,TMR.df$species)]
```

Summarize microclimate data

We also requried the mean and standard deviation of the hibernaculum temperature and water vapor deficit measurements.

```
#Read in microclimate measurement file
mc <- read.csv("microclimate.csv")

#Calculate summary statistics for entire hibernaculum
mc.summary.site <- mc %>%
  select_at(vars("Site.ID", "Ta_C", "RH_per", "dWVP_kPa")) %>%
  group_by(Site.ID) %>%
  summarise_all(c("min", "max", "mean", "sd"))
mc.summary.site <- as.data.frame(mc.summary.site)

#Summarize roosting location microclimate
mc.IBM <- data.frame()
for(s in 11:19){
  mc.species <- subset(mc, mc[,s]==TRUE)
  mc.species <- mc.species %>%
  select_at(vars("Ta_C", "RH_per", "dWVP_kPa")) %>%
  summarise_all(c("min", "max", "mean", "sd"))
  mc.species$species <- colnames(mc[s])
  mc.IBM <- rbind(mc.IBM, mc.species)
}
```

Determine predicted winter duration at each hibernaculum

Hranac et al., in review developed a model to predict the duration of winter at any location in North America. We used the output rasters from this model to extract the predicted duration of winter at each of our sampled hibernacula.

```r
library(raster)

#Read in site lat/long coordinates
sites <- read.csv("sites.csv")

#Read in winter duration prediction raster
dur  <- raster("durationRaster_p.tif")

#Extract winter duration predictions and attach to original site data frame
sites$Winter.Dur <- raster::extract(y = SpatialPoints(sites[,1:2]),x = dur,df=TRUE)[,2]
```

Run individual-based hibernation energetics model

The hibernation energetics model predicts fungal growth rate based on relationships between fungal growth and temperature and relative humidity as described by Hayman et al. 2016.

```r
#Read in fungal parameter data and select which model to use
fung.params <- read.csv("fungal_params.csv")
fung.params <- as.matrix(fung.params)
rownames(fung.params) <- c("Chaturvedi", "Verant")
fung.params <- fungalSelect("Chaturvedi")
```

We ran the hibernation energetics model for 100 bats within 100 winters for a total of 10,000 bat-runs of fat expenditure per species. **NOTE**: This will take a long time to run over all species.

```r
survival.ci     <- data.frame()
survival.winter <- data.frame()
survival.raw    <- data.frame()

#Run for all species
for(s in IBM$Species){
  df.species <- data.frame()

  #Run 100 "winters" with different hibernaculum conditions
  for(i in 1:100){
    df.merge <- data.frame()

    #Assign siteID and unique location ID
    siteID   <- params$SiteID[params$Species == toupper(s)]

    #Assign winter duration
    Wdur <- sites$Winter.Dur[sites$SiteID == (as.character(siteID))]

    #Create random "winter" climate from microclimate measurements
    temp.mean <- rnorm(1, mean = mc.IBM$Ta_C_mean[mc.IBM$species == s],
                          sd =   mc.IBM$Ta_C_sd[mc.IBM$species == s])

    rh.mean <- rnorm(1, mean = mc.IBM$RH_per_mean[mc.IBM$species == s],
                        sd =   mc.IBM$RH_per_sd[mc.IBM$species == s])

    #We assume all hibernaculum temperatures are above freezing
```

```r
temp.mean <- ifelse(temp.mean < 0, 0, temp.mean)

#We correct for the potential of relative humidity being greater than 100%
rh.mean <- ifelse(rh.mean > 100, 100, rh.mean)

#Create hibernaculum environment
env.df <- buildEnv(temp = temp.mean,
                   pct.rh = rh.mean,
                   range.res.temp = 1,
                   range.res.rh = 1,
                   twinter = 360,
                   winter.res = 24)

#Run 100 bats in hibernaculum
for(r in 1:100){
  #Read in parameter file for species
  s.params   <- as.list(params[params$Species == toupper(s),])

  #We assume a set proportion of body mass is fat and lean mass
  s.params$pFat  <- 0.26
  s.params$pLean <- 0.65

  #Replace parameters with random draws from a normal distribution
  s.params$Mass   <- rnorm(1, mean = IBM$Mass.Mean[IBM$Species  == s],
                              sd = IBM$Mass.SD[IBM$Species  == s])
  s.params$TMRmin <- rlnorm(1,meanlog = IBM$TMR.LMean[IBM$Species == s],
                              sdlog = IBM$TMR.LSD[IBM$Species == s])

  #Convert TMRmin to ml O2/h/g for use in model
  s.params$TMRmin <- s.params$TMRmin * 0.1793

  #Predict surface area given surface area scaling equations
  s.params$SA.body <- 10 * (s.params$Mass^(2/3))

  #Predict wing surface area as a proportion of body surface area
  #Determined from a photo analysis (described in Haase et al. 2019)
  s.params$SA.wing <- s.params$SA.body*s.params$Wing.prop

  #Run survival model
  surv.s <- hibernationModel(env = env.df,
                             bat.params = s.params,
                             fung.params = fung.params)

  #Summarize output and merge with previous runs
  df.surv <- data.frame(Species = toupper(s),
                        Winter  = i,
                        Bat     = r,
                        Ta      = temp.mean,
                        pct.rh  = rh.mean,
                        Mass    = s.params$Mass,
                        TMR     = s.params$TMRmin,
                        Max.null.days = max(surv.s$time[surv.s$surv.null==1])/24,
                        Max.inf.days  = max(surv.s$time[surv.s$surv.inf ==1])/24,
```

```
                            Null=ifelse(max(surv.s$time[surv.s$surv.null==1])/24>Wdur,1,0),
                            WNS =ifelse(max(surv.s$time[surv.s$surv.inf ==1])/24>Wdur,1,0))
      df.merge     <- rbind(df.merge, df.surv)
      survival.raw <- rbind(survival.raw,df.surv)
    }

    #Calculate "winter" survival %
    survival <- data.frame(Species = toupper(s),
                           Winter  = i,
                           Ta      = mean(df.merge$Ta),
                           pct.rh  = mean(df.merge$pct.rh),
                           Mass    = mean(df.merge$Mass),
                           TMR     = mean(df.merge$TMR),
                           Null    = sum(df.merge$Null)/100,
                           WNS     = sum(df.merge$WNS)/100)
    df.species      <- rbind(df.species, survival)
    survival.winter <- rbind(survival.winter, survival)
  }

  #Caclulate mean winter survival and confidence intervals for species
  survival.sp <- data.frame(Species   = toupper(s),
                            Ta        = mean(df.species$Ta),
                            Ta.sd     = sd(df.species$Ta),
                            pct.rh    = mean(df.species$pct.rh),
                            pct.rh.sd = sd(df.species$pct.rh),
                            Mass      = mean(df.species$Mass),
                            Mass.sd   = sd(df.species$Mass),
                            TMR       = mean(df.species$TMR),
                            TMR.sd    = sd(df.species$TMR),
                            Null      = mean(df.species$Null),
                            Null.lci  = mean(df.species$Null)-
                                        ((sd(df.species$Null)/
                                        sqrt(nrow(df.species)))*1.96),
                            Null.uci  = mean(df.species$Null)+
                                        ((sd(df.species$Null)/
                                        sqrt(nrow(df.species)))*1.96),
                            WNS       = mean(df.species$WNS),
                            WNS.lci   = mean(df.species$WNS)-
                                        ((sd(df.species$WNS)/
                                        sqrt(nrow(df.species)))*1.96),
                            WNS.uci   = mean(df.species$WNS)+
                                        ((sd(df.species$WNS)/
                                        sqrt(nrow(df.species)))*1.96),
                            Duration  = Wdur)

survival.ci <- rbind(survival.ci, survival.sp)
}
```

We want to relate the real, measured microclimate and morphometric data to predicted survival.

```
#Real microclimate data
survival.ci <- add_column(survival.ci,
              Ta.real = mc.IBM$Ta_C_mean[match(survival.ci$Species,
```

```
                                              toupper(mc.IBM$species))],
                .after = "Ta.sd")
survival.ci <- add_column(survival.ci,
                dWVP.real = mc.IBM$dWVP_kPa_mean[match(survival.ci$Species,
                                                    toupper(mc.IBM$species))],
                .after = "pct.rh.sd")

#Real morphometric and physiological data
survival.ci <- add_column(survival.ci,
                Mass.real = IBM$Mass.Mean[match(survival.ci$Species,
                                              toupper(IBM$Species))],
                .after = "Mass.sd")
survival.ci <- add_column(survival.ci,
                TMR.real = exp(IBM$TMR.Mean[match(survival.ci$Species,
                                                toupper(IBM$Species))]),
                .after = "TMR.sd")
survival.ci <- add_column(survival.ci,
                EWL.real = params$EWLg[match(survival.ci$Species,
                                           params$Species)],
                .after = "TMR.real")
```

Statistical analyses

We fit a linear model to the probability of survival with body mass, mass-specific torpid metabolic rate, mass-specific evaporative water loss, hibernaculum temperature, and hibernaculum water vapor deficit as predictors. We first test for correlated variables.

```
cor(survival.ci[,c("Ta.real","dWVP.real","Mass.real","TMR.real","EWL.real")])
```

```
##              Ta.real  dWVP.real   Mass.real    TMR.real   EWL.real
## Ta.real    1.0000000  0.3915829 -0.17814269 -0.35253705  0.3253057
## dWVP.real  0.3915829  1.0000000  0.26510906 -0.47023647 -0.6075292
## Mass.real -0.1781427  0.2651091  1.00000000  0.05108957 -0.3331781
## TMR.real  -0.3525371 -0.4702365  0.05108957  1.00000000  0.1731270
## EWL.real   0.3253057 -0.6075292 -0.33317806  0.17312705  1.0000000
```

We determined that the hibernaculum water vapor deficit and mass-specific evaportative water loss are correlated. We therefore fit two models and selected the model with the best fit.

```
summary(lm(WNS ~ scale(Ta.real,   center = T, scale = T) +
                 scale(Mass.real, center = T, scale = T) +
                 scale(TMR.real,  center = T, scale = T) +
                 scale(EWL.real,  center = T, scale = T),
        data = survival.ci))
```

```
##
## Call:
## lm(formula = WNS ~ scale(Ta.real, center = T, scale = T) + scale(Mass.real,
##     center = T, scale = T) + scale(TMR.real, center = T, scale = T) +
##     scale(EWL.real, center = T, scale = T), data = survival.ci)
##
```

```
## Residuals:
##        1        2        3        4        5        6        7        8
##  0.29110 -0.08020 -0.15975  0.04880 -0.09455 -0.01927 -0.02530 -0.02897
##        9
##  0.06814
##
## Coefficients:
##                                        Estimate Std. Error t value
## (Intercept)                             0.34263    0.06113   5.605
## scale(Ta.real, center = T, scale = T)    0.08152    0.07634   1.068
## scale(Mass.real, center = T, scale = T)  0.30767    0.06927   4.442
## scale(TMR.real, center = T, scale = T)  -0.03675    0.07358  -0.499
## scale(EWL.real, center = T, scale = T)  -0.08358    0.07622  -1.097
##                                        Pr(>|t|)
## (Intercept)                             0.00497 **
## scale(Ta.real, center = T, scale = T)    0.34577
## scale(Mass.real, center = T, scale = T)  0.01132 *
## scale(TMR.real, center = T, scale = T)   0.64370
## scale(EWL.real, center = T, scale = T)   0.33441
## ---
## Signif. codes:  0 '***' 0.001 '**' 0.01 '*' 0.05 '.' 0.1 ' ' 1
##
## Residual standard error: 0.1834 on 4 degrees of freedom
## Multiple R-squared:  0.8729, Adjusted R-squared:  0.7457
## F-statistic: 6.865 on 4 and 4 DF,  p-value: 0.04438
```

```
summary(lm(WNS ~ scale(Ta.real,   center = T, scale = T) +
                 scale(Mass.real, center = T, scale = T) +
                 scale(TMR.real,  center = T, scale = T) +
                 scale(dWVP.real, center = T, scale = T),
        data = survival.ci))
```

```
##
## Call:
## lm(formula = WNS ~ scale(Ta.real, center = T, scale = T) + scale(Mass.real,
##     center = T, scale = T) + scale(TMR.real, center = T, scale = T) +
##     scale(dWVP.real, center = T, scale = T), data = survival.ci)
##
## Residuals:
##         1         2         3         4         5         6         7
##  0.132356 -0.068195 -0.172932 -0.001647  0.025036  0.033753 -0.005659
##         8         9
##  0.012851  0.044438
##
## Coefficients:
##                                         Estimate Std. Error t value
## (Intercept)                             0.342634   0.039446   8.686
## scale(Ta.real, center = T, scale = T)   -0.001155   0.048525  -0.024
## scale(Mass.real, center = T, scale = T)  0.277728   0.046299   5.999
## scale(TMR.real, center = T, scale = T)  -0.005297   0.049027  -0.108
## scale(dWVP.real, center = T, scale = T)  0.156385   0.053662   2.914
##                                        Pr(>|t|)
## (Intercept)                            0.000967 ***
## scale(Ta.real, center = T, scale = T)   0.982143
```

```
## scale(Mass.real, center = T, scale = T) 0.003886 **
## scale(TMR.real, center = T, scale = T)  0.919172
## scale(dWVP.real, center = T, scale = T) 0.043493 *
## ---
## Signif. codes:  0 '***' 0.001 '**' 0.01 '*' 0.05 '.' 0.1 ' ' 1
##
## Residual standard error: 0.1183 on 4 degrees of freedom
## Multiple R-squared:  0.9471, Adjusted R-squared:  0.8941
## F-statistic: 17.89 on 4 and 4 DF,  p-value: 0.008113
```

We determined that the best fit model is that with the hibernaculum water vapor deficit.

To determine the contribution of each covariate to describing variation in survival, we calculated the squared semi-partial correlation coefficient (SPCC), which is the squared correlation (r^2) between the variable in question (X_i) and the prediction of the response variable ($\hat{y}$) without the influence of the other variable (Kim 2015).

```
library(ppcor)
spcor(survival.ci[,c("WNS","Ta.real","dWVP.real","Mass.real",
                     "TMR.real","EWL.real")])$estimate[1,]^2
```

```
##          WNS      Ta.real    dWVP.real    Mass.real     TMR.real
## 1.0000000000 0.0091150091 0.0896809774 0.4699296494 0.0001354324
##     EWL.real
## 0.0154879794
```

```
pcor(survival.ci[,c("WNS","Ta.real","dWVP.real","Mass.real",
                    "TMR.real","EWL.real")])$estimate[1,]^2
```

```
##         WNS     Ta.real   dWVP.real   Mass.real    TMR.real    EWL.real
## 1.000000000 0.195715596 0.705379090 0.926175304 0.003602581 0.292525741
```

We used a Kruskal-Wallace test (for non-parametric data) to test for differences in survival with WNS among bat species and to determine significant pairwise relationships between species.

```
library(pgirmess)
kruskal.test(WNS ~ Species, data = survival.winter)
```

```
##
##  Kruskal-Wallis rank sum test
##
## data:  WNS by Species
## Kruskal-Wallis chi-squared = 529.6, df = 8, p-value < 2.2e-16
```

```
kruskalmc(WNS ~ Species, data = survival.winter)
```

```
## Multiple comparison test after Kruskal-Wallis
## p.value: 0.05
## Comparisons
##           obs.dif critical.dif difference
## COTO-EPFU 172.480     117.5287       TRUE
```

```
## COTO-MYCI 348.305     117.5287       TRUE
## COTO-MYEV 327.935     117.5287       TRUE
## COTO-MYLU 288.320     117.5287       TRUE
## COTO-MYTH 238.205     117.5287       TRUE
## COTO-MYVE  82.380     117.5287      FALSE
## COTO-MYVO 271.830     117.5287       TRUE
## COTO-PESU 137.510     117.5287       TRUE
## EPFU-MYCI 520.785     117.5287       TRUE
## EPFU-MYEV 500.415     117.5287       TRUE
## EPFU-MYLU 460.800     117.5287       TRUE
## EPFU-MYTH 410.685     117.5287       TRUE
## EPFU-MYVE  90.100     117.5287      FALSE
## EPFU-MYVO 444.310     117.5287       TRUE
## EPFU-PESU 309.990     117.5287       TRUE
## MYCI-MYEV  20.370     117.5287      FALSE
## MYCI-MYLU  59.985     117.5287      FALSE
## MYCI-MYTH 110.100     117.5287      FALSE
## MYCI-MYVE 430.685     117.5287       TRUE
## MYCI-MYVO  76.475     117.5287      FALSE
## MYCI-PESU 210.795     117.5287       TRUE
## MYEV-MYLU  39.615     117.5287      FALSE
## MYEV-MYTH  89.730     117.5287      FALSE
## MYEV-MYVE 410.315     117.5287       TRUE
## MYEV-MYVO  56.105     117.5287      FALSE
## MYEV-PESU 190.425     117.5287       TRUE
## MYLU-MYTH  50.115     117.5287      FALSE
## MYLU-MYVE 370.700     117.5287       TRUE
## MYLU-MYVO  16.490     117.5287      FALSE
## MYLU-PESU 150.810     117.5287       TRUE
## MYTH-MYVE 320.585     117.5287       TRUE
## MYTH-MYVO  33.625     117.5287      FALSE
## MYTH-PESU 100.695     117.5287      FALSE
## MYVE-MYVO 354.210     117.5287       TRUE
## MYVE-PESU 219.890     117.5287       TRUE
## MYVO-PESU 134.320     117.5287       TRUE
```

We then used a Tukey Honest Significant Difference test to determine differences between mass-specific metabolic rate, mass-specific evaporative water loss, body mass, and the hibernaculum microclimate. Because of the large sample size, we subset the data to less samples per species.

```
m.df <- data.frame(do.call("rbind", by(morph, morph["species"], head, n=30)))
TukeyHSD(aov(log(m.df$capture.mass) ~ m.df$species))

r.df <- resp.rm[resp.rm$nom.temp == 8,]
r.df <- data.frame(do.call("rbind", by(r.df, r.df["species"], head, n=30)))
TukeyHSD(aov(log(r.df$tmr.mw.g) ~ r.df$species))
TukeyHSD(aov(log(r.df$ewlg) ~ r.df$species))

mc.df <- data.frame(do.call("rbind", by(mc, mc["Site.ID"], head, n=30)))
TukeyHSD(aov(mc.df$dWVP_kPa ~ mc.df$Site.ID))
TukeyHSD(aov(mc.df$Ta_C ~ mc.df$Site.ID))
```

Literature Cited

Haase CG, Fuller NW, Hranac CR, Hayman DTS, McGuire LP, Norquay KO, Silas KA, Willis CKR, Plowright RK, Olson SH (2019) Incorporating evaporative water loss into bioenergetic models of hibernation to test for relative influence of host and pathogen traits on white-nose syndrome. PLOS ONE 14(10):e0222311.

Hayman DTS, Pulliam JRC, Marshall JC, Cryan PM, Webb CT (2016) Environment, host, and fungal traits predict continental-scale white-nose syndrome in bats. Science Advances 2(1):e1500831.

Kim S (2015) ppcor: An R Package for a Fast Calculation to Semi-partial Correlation Coefficients. Communications for Statistical Applications and Methods 22(6):665-674.
