# Supplementary material for: Body mass and hibernation microclimate may predict bat susceptibility to white‐nose syndrome
Source: Ecol Evol. 2020 Dec 21;11(1):506–15. doi: 10.1002/ece3.7070 (PMC7790633; doi:10.1002/ece3.7070)
Supplement: Supplementary file 2 — Supplement S2 [file ECE3-11-506-s002.docx]

**Supplementary Materials**

We captured nine bat species from eight sites during pre-hibernation (September – November) and during mid-hibernation (January-March) of 2016-2019 (Tables 1-2, Figure 1). At each site, we caught bats using mist nets and harp traps at the hibernaculum entrance during pre-hibernation and hand-captured from hibernaculum walls during mid-hibernation.

Bats were weighed (±0.1 g) before placing them in a chamber and running them in open-flow respirometry (Lighton 2008) to measure torpid metabolic rate (TMR) and evaporative water loss (EWL) across multiple temperatures (see Haase et al. 2019 for complete methodology). We placed bats in individual chambers in a dark temperature-controlled cabinet at 10°C for approximately 12 hours until bats became torpid. We then reduced chamber temperatures to 8°C, 5°C, and 2°C for three hours each to ensure bats reached steady-state at each ambient temperature. Respirometry flow rate was controlled with an airflow manifold (FB-8; Sable Systems) and we used a multiplexer (RM-8; Sable Systems) to continuously rotate among bats for the duration of the respirometry measurements. We maintained dry air at 0% relative humidity by scrubbing with magnesium perchlorate and measured excurrent water vapor pressure with a humidity meter (RH-300; Sable Systems). We calculated metabolic rate from CO_2_ production and water vapor density (WVD) from water vapor pressure using equations from Lighton (2008). We converted CO_2_ values to mW using the energetic equivalent 28.008 ml CO_2_ J^-1^. As all measurements were made after at least 12 h of settling time, we assumed all bats were post-absorptive (Boratyński et al. 2015). We then calculated EWL (mg H_2_O h^-1^) by multiplying WVD (µg/ml) by the flow rate (mL min^-1^) and then converted this to mg h^-1^ by multiplying by 0.06.

We classified bats to be torpid when O_2_ consumption measurements were below 0.35 ml O_2_ h^-1^ g^-1^, a threshold determined from an exhaustive literature search (Blood & Dodgen, 1956; Boratyński et al., 2015; Day & Tomasi, 2014; Dunbar, 2007; Dunbar & Brigham, 2010; Geiser, 2004; Hock, 1951; B. J. Klüg-Baerwald et al., 2017; McGuire et al., 2017; Riedesel & Williams, 1976; Speakman & Thomas, 2003; Thomas, Cloutier, & Gagné, 1990; Willis, Turbill, & Geiser, 2005; Wojciechowski, Jefimow, & Tęgowska, 2007). We then defined the temperature range of minimum torpid metabolic rates for each species by fitting a linear mixed effects model between O_2_ consumption and temperature (with inclusion of site, season, and a random effect of individual bat). In the event that there was an effect of temperature, we used a Games-Howell post-hoc comparison to determine which temperatures were statistically different. We defined those that were not different as the range of minimum torpid metabolic rates and used the lowest of these temperatures as the minimum defended temperature in torpor. We took the EWL measurements associated with these metabolic rate measurements. Finally, we calculated the mean area-specific rate of evaporative water loss for each species given the measured EWL, water vapor pressure, and mean surface area calculated as described in Haase et al. (2019).

We measured temperature and relative humidity of roosts within each hibernaculum using HOBO (± 0.45°C, ± 2.5% relative humidity; Model U23-001, Onset Computer Corporation) and iButton (± 1°C; Model DS1921Z-F5, Maxim Integrated Products) data loggers. During pre-hibernation, we placed four HOBO and ten iButton loggers throughout each hibernaculum recording at 3 h intervals. We collected loggers after hibernation each year and converted relative humidity to water vapor deficit using equations in Campbell and Norman (1998). We then calculated water vapor deficit as the difference between saturation and measured water vapor pressure (kPa) given relative humidity and air temperature.

Supplementary Table S1. Results of comparisons across bat species. Differences in the probability of survival through hibernation with white-nose syndrome (WNS) were tested with a Kruskall-Wallis rank sum test (critical difference = 117.53; observed reported). All other comparisons, including mass-specific torpid metabolic rate (TMR), mass-specific evaporative water loss (EWL), hibernaculum water vapor deficit (dWVP) and temperature (T_a_), were with a Tukey’s Honest Significant Difference test (p-values reported).

| Species | WNS  Survival | Mass (g) | TMR  (ml O_2_ h^-1^ g^-1^) | EWL  (mg H_2_O h^-^1 g^-1^) | dWVP (kPa) | T_a_  (°C) |
| --- | --- | --- | --- | --- | --- | --- |
| COTO-EPFU | 172.48 | <0.0001 | 0.939 | 0.990 | 0.886 | <0.0001 |
| COTO-MYCI | 348.31 | <0.0001 | 0.431 | 0.990 | NA | NA |
| COTO-MYEV | 327.94 | <0.0001 | 0.990 | 0.035 | <0.0001 | 0.044 |
| COTO-MYLU | 288.32 | <0.0001 | 0.654 | 0.003 | <0.0001 | 0.044 |
| COTO-MYTH | 238.21 | 0.586 | 0.732 | 0.206 | <0.0001 | 0.044 |
| COTO-MYVE | 82.38 | <0.0001 | 0.247 | 0.364 | <0.0001 | <0.0001 |
| COTO-MYVO | 271.83 | 0.018 | 0.834 | 0.539 | <0.0001 | 0.044 |
| COTO-PESU | 137.51 | <0.0001 | 0.073 | 0.470 | <0.0001 | <0.0001 |
| EPFU-MYCI | 520.79 | <0.0001 | 0.990 | 0.990 | 0.886 | <0.0001 |
| EPFU-MYEV | 500.42 | <0.0001 | 0.965 | 0.105 | 0.002 | <0.0001 |
| EPFU-MYLU | 460.80 | <0.0001 | 0.992 | 0.017 | 0.002 | <0.0001 |
| EPFU-MYTH | 410.69 | <0.0001 | 0.989 | 0.357 | 0.002 | <0.0001 |
| EPFU-MYVE | 90.10 | 0.002 | 0.149 | 0.519 | 0.052 | 0.002 |
| EPFU-MYVO | 444.31 | <0.0001 | 0.987 | 0.663 | 0.002 | <0.0001 |
| EPFU-PESU | 309.99 | <0.0001 | 0.990 | 0.626 | 0.052 | 0.002 |
| MYCI-MYEV | 20.37 | 0.185 | 0.805 | 0.095 | <0.0001 | 0.006 |
| MYCI-MYLU | 59.99 | 0.043 | 0.987 | 0.009 | <0.0001 | 0.006 |
| MYCI-MYTH | 110.10 | <0.0001 | 0.990 | 0.421 | <0.0001 | 0.006 |
| MYCI-MYVE | 430.69 | <0.0001 | 0.009 | 0.631 | <0.0001 | <0.0001 |
| MYCI-MYVO | 76.48 | <0.0001 | 0.990 | 0.795 | <0.0001 | 0.006 |
| MYCI-PESU | 210.80 | 0.185 | 0.998 | 0.750 | <0.0001 | <0.0001 |
| MYEV-MYLU | 39.62 | 0.994 | 0.971 | 0.990 | NA | NA |
| MYEV-MYTH | 89.73 | <0.0001 | 0.868 | 0.990 | NA | NA |
| MYEV-MYVE | 410.32 | <0.0001 | 0.754 | 0.989 | 0.998 | <0.0001 |
| MYEV-MYVO | 56.11 | <0.0001 | 0.907 | 0.973 | NA | NA |
| MYEV-PESU | 190.43 | 0.998 | 0.499 | 0.943 | 0.998 | <0.0001 |
| MYLU-MYTH | 50.12 | <0.0001 | 0.995 | 0.858 | NA | NA |
| MYLU-MYVE | 370.70 | <0.0001 | 0.014 | 0.723 | 0.998 | <0.0001 |
| MYLU-MYVO | 16.49 | 0.017 | 0.990 | 0.656 | NA | NA |
| MYLU-PESU | 150.81 | 0.998 | 0.720 | 0.507 | 0.998 | <0.0001 |
| MYTH-MYVE | 320.59 | <0.0001 | 0.045 | 0.989 | 0.998 | <0.0001 |
| MYTH-MYVO | 33.63 | 0.795 | 0.990 | 0.990 | NA | NA |
| MYTH-PESU | 100.70 | <0.0001 | 0.998 | 0.987 | 0.998 | <0.0001 |
| MYVE-MYVO | 354.21 | <0.0001 | 0.085 | 0.990 | 0.998 | <0.0001 |
| MYVE-PESU | 219.89 | <0.0001 | <0.0001 | 0.987 | NA | NA |
| MYVO-PESU | 134.32 | <0.0001 | 0.990 | 0.992 | 0.998 | <0.0001 |
